# Glycogen depletion in astrocytes induces sex-dimorphic remodeling of astrocytic and synaptic structures with concomitant anxiety-like behaviors and maternal care deficits

**DOI:** 10.1186/s13293-025-00723-6

**Published:** 2025-06-11

**Authors:** Xiaotong Shi, Yuanyuan Zhu, Zhaoyichun Zhang, Ningcan Ma, Danyi He, You Wu, Ziyi Dai, Xinyan Qin, Yingyi Chen, Youyi Zhao, Haopeng Zhang, Jing Huang, Hui Zhang, Ze Fan

**Affiliations:** 1 State Key Laboratory of Oral & Maxillofacial Reconstruction and Regeneration, National Clinical Research Center for Oral Diseases, Shaanxi Engineering Research Center for Dental Materials and Advanced Manufacture, Department of Anesthesiology, School of Stomatology, The Fourth Military Medical University, Xi’an, Shaanxi China; 2 Department of Neurobiology and Institute of Neurosciences, School of Basic Medicine, The Fourth Military Medical University, Xi’an, Shaanxi China; 3 Department of Stomatology, Xi’an Medical University, Xi’an, Shaanxi China

**Keywords:** Glycogen, Anxiety, Maternal behavior, Structural plasticity, Sex differences

## Abstract

**Background:**

Maternal care is an instinctive social behavior indispensable for survival and gene transmission. Postpartum maternal behavior is profoundly affected by mother’s emotional state via incompletely elucidated complex mechanisms including metabolic regulation. Brain glycogen, primarily located in astrocytes, is a potent modulator for brain plasticity and provides neuroprotection against bioenergetic insults. The regulation of brain glycogen is of relevance to hormonal control that might be linked to sex-dimorphic responses in mental health. The present study aims to investigate the involvement of glycogen in the sex differences of brain structural plasticity, and to characterize the impacts on affective and maternal behaviors in both sexes.

**Methods:**

Male and female brain-type glycogen phosphorylase knock-in (Pygb-KI) mice were generated to exhaust glycogen in astrocytes in both sexes. Metabolomics, seahorse and relative assay kits were utilized to detect the changes in downstream metabolites to assess the effects of astrocytic glycogen depletion on energy metabolism. Virus-labeling, immunostaining combined with sholl analysis were performed to explore the morphological changes in astrocytes, neurons and dendrite spines. In addition, affective behaviors were assessed using the open field and elevated plus maze tests to quantify anxiety-like phenotypes, and the tail suspension test to evaluate depressive-like components of behavior. Maternal care was analyzed through pup retrieval assays and nest-building behavior, while offspring development was tracked via survival rates and ultrasonic vocalizations. Expression of hormonal receptors was identified via qPCR and immunofluorescence staining.

**Results:**

Pygb-KI mice exhibited glycogen deficiency in astrocytes in both sexes, causing disrupted energy metabolic patterns, particularly in glycolysis. Subsequently, we observed in female-specific decreases in area, branching, and length of astrocytes and loss of mature dendritic spines in neurons. This sex-dimorphic phenotype was in accordance with the phenomenon that Pygb-KI females displayed anxiety-like behaviors in adulthood, irrespective of the virgin or lactating stage. Assessment of maternal behaviors revealed that Pygb-KI lactating mice displayed maternal care obstacles, and offspring nursed by Pygb-KI dams showed reduced survival rate and social deficits during development. Estradiol signaling was attenuated while glucocorticoid signaling was elevated in Pygb-KI females during the lactation stage.

**Conclusion:**

Our findings demonstrate that astrocytic glycogen depletion induces female-specific disruption of structural plasticity in astrocytes and synapses, with these morphological alterations correlating with sex-dimorphic abnormalities in anxiety-like and maternal behaviors. These results reveal a sexually dimorphic mechanism whereby astrocytic glycogen loss selectively impairs structural plasticity in females, thereby underscoring the critical role of glycogen homeostasis in female-specific neurobehavioral adaptations essential for species survival.

**Supplementary Information:**

The online version contains supplementary material available at 10.1186/s13293-025-00723-6.

## Highlights

Glycogen deficiency in astrocytes correlates with glycolysis decrement in Pygb-KI mice.

Pygb-KI mice display female-specific decreases in area, branching, and length in astrocytes, and loss of mature dendritic spines in pyramidal neurons.

Glycogen depletion in astrocytes shows a female-biased association with anxiety-like behaviors, whereas depressive-like phenotypes remain unaffected across sexes.

Glycogen depletion in astrocytes is associated with maternal care obstacles, reduced survival rate and social communication ability of offspring, and disruptive hormonal signaling pathways in lactating mice.

Glycogen depletion in astrocytes triggers female-restricted remodeling of astrocytic and synaptic structures which correlate with exclusively female behavioral impairments.

**Supplementary Information:**

The online version contains supplementary material available at 10.1186/s13293-025-00723-6.

## Plain language summary

Maternal care is a sex-dimorphic behavior that is necessary for survival and reproduction in species. At present, postpartum anxiety, depression as well as maternal care obstacles have become serious social problems that have garnered increasing public attention. It’s known that metabolic disorders in the brain are closely associated with mental illnesses. Brain glycogen serves as the largest on-demand energy source within the brain, predominantly stored in astrocytes and acts as a necessary component for synaptic formation and plasticity. Whether brain glycogen metabolism contributes to sex differences in emotional and maternal behaviors is unknown. Here, by generating a glycogen depletion model via a transgenic brain-type glycogen phosphorylase knock-in (Pygb-KI) method, we observed glycolysis decrement in both sexes. Furthermore, female-specific decreases in area, branching, and length in astrocytes, as well as loss of mature dendritic spines in pyramidal neurons were observed compared to male mice. Additionally, female Pygb-KI mice displayed anxiety-like behaviors in both virgin and reproductive periods. We also discovered maternal care deficits, reduced survival rate and social communication ability in pups, and dysregulations in neuronal hormonal signaling pathways in Pygb-KI lactating mice. These results may provide a perspective that targeting astrocytic glycogen metabolism may supply effective therapeutics for addressing postpartum anxiety and maternal behavioral deficits in clinical settings. But additional work is needed to investigate how astrocytic glycogen depletion triggers the neuronal plasticity and maternal behavioral phenotypes in females.

**Supplementary Information:**

The online version contains supplementary material available at 10.1186/s13293-025-00723-6.

## Introduction

The transition to motherhood represents a pivotal event for women, encompassing both physiological and psychological dimensions [1]. An appropriate maternal response is essential, yet poses a challenge to achieve, for the survival and normal development of mammalian newborns, including humans [2]. It requires caretakers to maintain a well-balanced diet, sufficient rest, regular physical activity, and especially, stable emotional health, to maximize the probability of successful reproduction and offspring maturity [3]. In the past decades, the growth of postpartum anxiety and depression has garnered increasing public attention, which driving the studies uncovering the relationships among sex differences in emotional distress, parental care obstacles as well as offspring developmental behavioral anomalies [4]. Nevertheless, the neurobiological substrates mediating maternal affective processes and dyadic regulatory dysfunction in early caregiving relationships have yet to be fully elucidated.

In preparation for challenges of gestation, parturition and lactation, the maternal brain undergoes vast metabolic modifications that shape and adapt it throughout the pregnancy period [5]. These adaptations of energy re-balance and metabolic re-homeostasis are required for optimal development of infants and can regulate neuronal circuits and plasticity, ultimately leading to long-term behavioral changes in dams [6]. Glycogen serves as the largest on-demand energy source within the brain, predominantly stored in astrocytes [7]. It can be rapidly degraded into monosaccharide via glycogen phosphorylase to provide energy for surrounding neurons when lacking energy supply [8]. According to literature, glycogen in astrocytes acts as a necessary component for synaptic formation and plasticity, and exerts sensitive responses to hormonal fluctuations, such as estrogen and glucocorticoids [9, 10]. Mutual regulation exists between hormone profiles and glycogen metabolism in the central nervous system (CNS) [11]. Specifically, estradiol is reported to increase astrocyte glycogen content in female but not in male astrocytes, with female astrocytes displaying greater sensitivity to estradiol stimulation in terms of glycogen metabolic enzyme profiles [12]. Disruptions in astrocytic glycogen metabolism result in harmful impacts on neurophysiology across multiple levels, encompassing astrocyte-neuron interactions, neuronal excitability, and cognitive behaviors [13, 14]. However, whether glycogen is involved in the sex differences of brain structural plasticity, and the impacts on affective and maternal disorders between sexes is still lack of investigation.

In current study, by generating astrocytic specific brain type glycogen phosphorylase knock in (Pygb-KI) mice, we produced a model of glycogen depletion in mice’s brain of both sexes. Integrating metabolomics and energy metabolism analyses revealed that the productions of glycogenolysis as well as the capacity of glycolysis were reduced in astrocytes. Sholl analysis revealed the decreases in area, branching, and length in astrocytes, and loss of mature dendritic spines in female but not male mice. Additionally, we identified anxiety-like behaviors in adult Pygb-KI females compared with wild type (WT) mice. These behavioral phenotypes could sustain to the lactation period, and are associated with aberrant maternal behaviors in lactating mice. Afterwards, we observed less activated oxytocin receptor (OXTR) positive neurons in the region of medial preoptic area (MPOA) in KI-dams when taking care of their pups, and disruptions in neuronal hormonal signaling pathways at the lactation stage. These results indicated disrupted neural basis of maternal behavior in Pygb-KI females. Consequently, offspring nursed by KI-dams showed reduced survival rate and social deficits during childhood and adolescence. Collectively, our data reveal that excessive glycogenolytic programs with astrocytic glycogen deficiency in the brain are associated with abnormalities in morphological development of astrocytes as well as maturity of synapses in a sex-dependent manner. These neuroglial remodeling patterns are linked to anxiety-like behavioral signatures and maternal care deficits specifically observed in female cohorts. Targeting on astrocytic glycogen metabolism may supply effective therapeutics for addressing postpartum anxiety and maternal behavioral deficits in clinical settings.

## Methods

### Animals

C57BL/6 mice were obtained from the Laboratory Animal Center of the Fourth Military Medical University. Pygb-KI transgenic mice were generated via CRISPR/Cas9-mediated homology-directed repair (HDR) from Cyagen Biosciences Inc. (Guangzhou, China). Briefly, a plasmid containing GFAP promotor, mouse Pygb cDNA, and polyadenylation signal was injected into pronuclei of C57BL/6 zygotes. Germline transmission was confirmed by breeding PCR-positive founders with wild-type littermates, resulting in F1 progeny. These F1 mice were then interbred to produce homozygous F2 mice for subsequent studies. All animals utilized in this study were housed in a standardized environment with a temperature ranging from 22 to 25 °C, a humidity of 50% and a 12-h/12-h light/dark cycle, and were treated by the guideline of the Care and Use of Laboratory Animals published by the National Institutes of Health (NIH). C57BL/6J and Pygb-KI virgin mice were maintained in genotype-segregated housing under standardized conditions. Each mixed-sex cohort (3 males and 3 females) was group-housed in a single cage, with strict separation between wild-type and transgenic populations throughout the study. Daily vaginal plug checks identified successfully bred females, which were individually housed 4 days prepartum to minimize social stress during late gestation. All procedures were approved by the Institutional Animal Care and Use Committee of the Fourth Military Medical University.

### Glycogen measurement

**Fig. 1:**
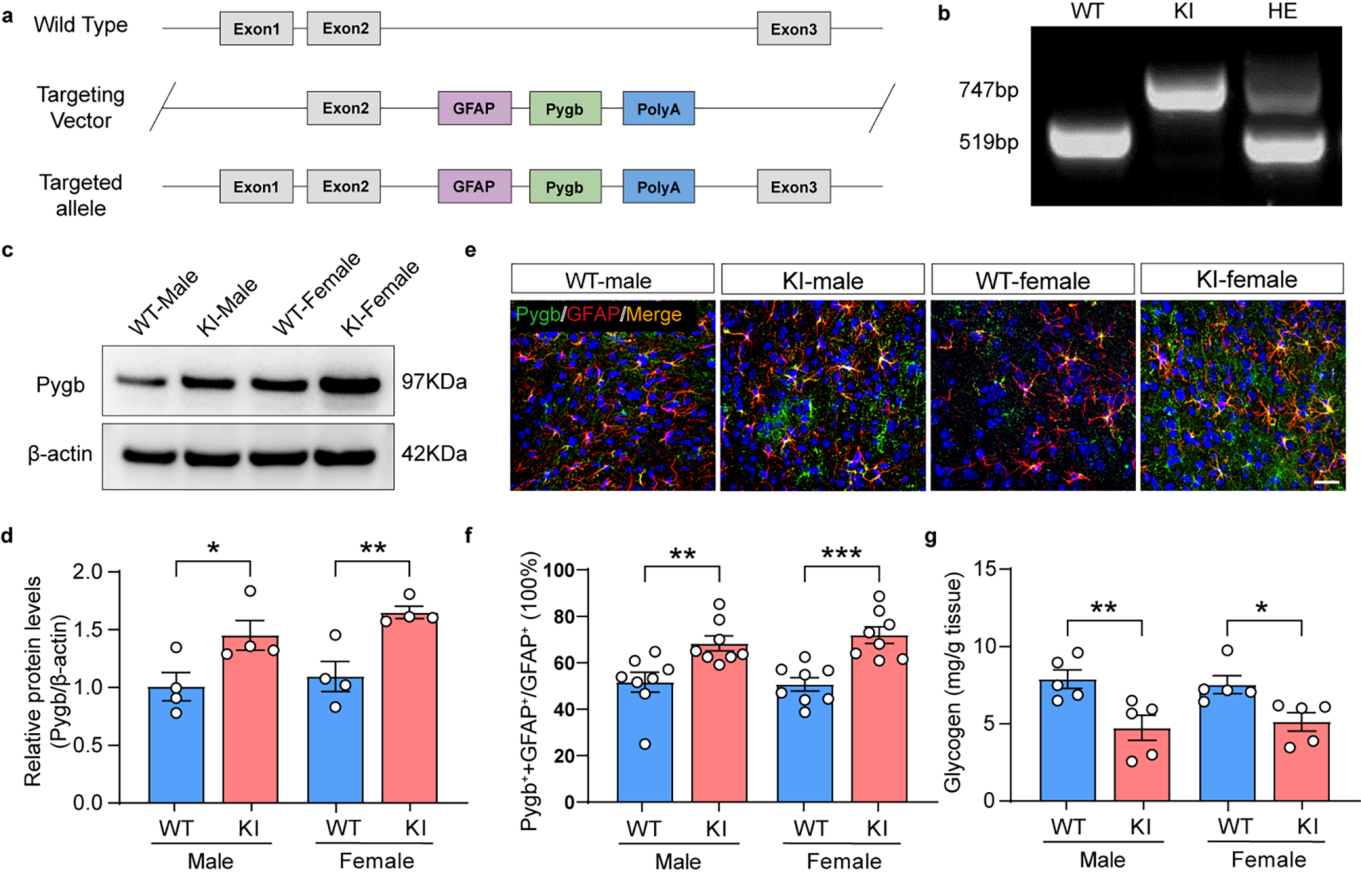
Enhanced glycogenolytic programs and glycogen deficiency in astrocyte specific Pygb-KI mice. (**a**) Schematic diagram of the CRISPR-/Cas-mediated genome strategy for creating GFAP-specific Pygb knock-in mice. (**b**) Identification of WT and Pygb-KI mice using PCR on tail DNA samples. The positions and sizes of PCR fragments for wild-type and mutant mice are indicated. (**c-d**) Western blot (**c**) and quantification (**d**) of PYGB protein levels. (**e-f**) Representative images (**e**) and quantification (**f**) of the co-expression of Pygb (green) and GFAP (red) in the ACC of adult WT and Pygb-KI mice to ensure precise localization within astrocytic populations. Scale bars = 50 μm. (**g**) Glycogen level in WT and Pygb-KI mice. The data are denoted as the mean ± SEM. *P < 0.05, **P < 0.01, ***P < 0.001

WT and Pygb-KI mice aged 8–12 weeks were bred as described above. WT mice were progeny of separately maintained C57BL/6 colonies, whereas the Pygb-KI mice were homozygous F2 offspring obtained through hybridization, as validated by triplex PCR (Fig. 1b). The anterior cingulate cortex (ACC) of adult mice (*n* = 5 per genotype) received high-energy microwave to prevent glycogen breakdown via the focused microwave irradiation system (ORW1.5 S-Focus, Orient Microwave, China) as previously described [15]. Then, we obtained the brain tissue using a stereomicroscope (SZ51, Olympus, Japan) after decapitating the mice. A Glycogen Assay Kit (K648, BioVision, USA) was used to measure Glycogen content follow the manufacturer’s instructions.

### Metabolome profiling analysis

Brain tissues of the ACC of adult mice (8–12 weeks old) were harvested (*n* = 4 per group, regardless of genders) for metabolome profiling analysis conducted by Metware Co, Ltd (Wuhan, China). In brief, 50 mg tissues were treated with precooled 70% methanol water extract and then vortexed for 3 min. Then, the mixture was centrifuged twice at 12,000 g for 10 min each at 4 °C and the supernatants were collected. The samples were analyzed using the Ultra Performance Liquid Chromatography coupled with Tandem Mass Spectrometry. The metabolites were separated using mobile phase A and mobile phase B. Mobile phase A is an acetonitrile solution with 0.3% ammonia water and 10 mM ammonium acetate, and mobile phase B is an aqueous solution with 90% acetonitrile. The flow rate was 0.4 ml/min at a temperature of 40 °C. The optimized gradient elution procedure was as follows: 5% A (0–1.2 min), increased to 30% A (8 min), 50% A (9–11 min), finally back to 5% A (11.1–15 min), injection volume: 2 µl. Mass data acquisition was performed using a triple quadrupole-linear ion trap mass spectrometer (QTRAP^®^ 6500 + LC–MS/MS System, ABsciex, USA). Analyst software (1.6.3 version, ABsciex) and Multiquant software (3.0.3 version, ABsciex) were used to acquire and quantify all metabolites. Using Multiple Reaction Monitoring model to analyze quantitatively the results of mass spectrometry detection. Principal component analysis (PCA), orthogonal partial least-square discriminant analysis (OPLS-DA) and partial least-square discriminant analysis were used to determine the distributions and to find the metabolic differences between WT and KI groups.

### Glycolysis analysis

Glycolysis analysis was performed as mentioned in a previous report [16]. Briefly, primary astrocytes were isolated from neonatal WT and Pygb-KI mice (postnatal day 1–3, 6 wells per genotype) via mechanical dissociation and plated in poly-D-lysine coated 24-well XF assay plates at 60,000 cells/well to measure extracellular acidification rate (ECAR), an important index to evaluate the acidic substances produced by anaerobic fermentation during cell metabolism to reflect the degree of glycolysis [17], using a Seahorse extracellular flux analyzer (XF-24, Seahorse Bioscience, USA). After baseline measurements, the following reagents prepared in the assay medium were sequentially injected into each well: 10 mM glucose (47249, Sigma, USA), 0.5 µM oligomycin A (75351, Sigma), 50 mM 2-deoxy-d-glucose (D6134, Sigma). Cell number was counted base on DAPI staining (1:1,000, D9564, Sigma) after measurement for normalizing the ECAR value.

### Labeling and morphological 3D reconstruction of neural cells

#### Astrocytes

AAV2/5-GfaABC1D-EGFP-WPRE-pA (200 nl, OBiO Technology Inc. China) was stereotaxically delivered into bilateral ACC of 8-week-old mice using a micro-syringe pump (788130, RWD, China). Target coordinates relative to bregma were: AP + 1.00 mm, ML ± 0.40 mm, DV -1.50 mm. Brain slices were collected three weeks after injection for astrocyte analysis.

For 3D reconstruction, an Olympus FV3000 confocal microscope and Imaris 9.3.1 software (Bitplane) were used as reported previously [18]. The astrocytes were selected randomly from at least 6 slices in the prefrontal cortex of three WT or Pygb-KI mice respectively for analysis.

#### Neurons

The pyramidal neurons were transfected by virus of AAV_PHP.eB_-hSyn1-EGFP-P2A-EGFPf (PackGene Biotech Inc. China) via retro-ocular injection as mentioned earlier [19]. In brief, after anesthetizing mice (8–12 weeks old) with 3% isoflurane, 100 µl of virus were injected into the retro-orbital sinus. The mice were kept in their cages for three weeks then transcranially perfused to collect slices for neuronal analysis.

Using Olympus FV3000 confocal microscope Imaris 9.3.1 software (Bitplane) to create three-dimensional reconstructions. The spine was classified as bellow: stubby: length (spine) < 1 μm; mushroom: length > 3 μm and max width (head)/mean width (neck) > 2; long thin: mean width (head)/mean width (neck) ≥ 1; and the rest were filopodia. The data for sholl analysis were obtained from 3 sections per mouse, while for spine analysis were gathered from 5 neurons per section, in each group of mice consisting of three mice in total.

### Western blotting

The ACC tissues of adult mice (8–12 weeks old) were dissected and homogenized in RIPA lysis buffer containing protease (43002700, Roche, Switzerland) and phosphatase inhibitors (41659200, Roche). The protein samples were quantified using a BCA Protein Assay Kit (23335, Thermo, USA), separated by 10% SDS–PAGE and transferred to PVDF membrane (C228814, Millipore, USA). Subsequently, the membranes were incubated with 5% (w/v) skim milk for 2 h, followed by overnight incubation with the primary antibodies as listed in Table S1 at 4 °C. After washing with TBST, the membranes were incubated with HRP-conjugated secondary antibodies at room temperature for 2 h. Proteins were imaged by chemiluminescence imaging system (5200 Multi, Tanon, China) and analyzed by ImageJ software (version 1.48). Antibody validation data were provided in Table S1.

### Immunofluorescence staining

After anesthetized by pentobarbital (50 mg/kg, i.p. injection), mice (8–12 weeks old) were undergoing transcardiac perfusion with 30 ml of precooled 0.01 M PBS, followed by 40 ml of precooled 4% (w/v) paraformaldehyde (PFA). Serial 30 μm coronal sections encompassing the ACC (bregma + 0.30 to + 1.50 mm according to Paxinos & Watson, 7th ed.) were obtained using a cryostat microtome (CM1905, Leica) maintained at -20 °C. The brains were removed and sectioned into 30 μm thick slices on a freezing microtome (CM1905, Leica, Germany) after immersed in 30% (w/v) sucrose for an additional 48 h. The brain sections were blocked by PBS containing 3% BSA (A1933, Sigma) and 0.3% Triton-X100 (T9284, Sigma) and then incubated with primary and secondary antibodies listed in Table S1 in sequence. All sections were incubated with DAPI (1:1,000, D9564, Sigma) for 15 min and then imaged under a confocal microscope (Fluoview Ver4.2 b, Olympus).

### Quantitative real-time PCR

Total RNA was extracted from the ACC of adult mice (8–12 weeks old) with TRIzol reagent (15596018, Ambion) and then quantified using a NanoDrop spectrophotometer (ND-NDL-2YRW-CCC, TSF). cDNA was synthesized from 2 µg of total RNA using the Prime Script RT Reagent Kit (RR036A, TaKaRa) and then amplified using SYBR Green Master Mix (B21202, Bimake) on a CFX96 PCR system (Bio-Rad Laboratories, USA). The values were normalized based on the expression of β-actin as a reference. Primer sequences were listed in Table S2.

### Behavioral tests

Consecutive behavioral assessments (open field test, elevated plus maze test, tail suspension test) were administered to identical postpartum/lactating cohorts using a counterbalanced randomization sequence with 24-hour inter-test intervals to eliminate stress carryover effects [20]; Resident-intruder assays employed independent cohorts housed in individually ventilated cages to preclude social interaction artifacts; Maternal care behavior and pup retrieval tests shared dams, while ultrasonic vocalization utilized their PND9 offspring. The details were suppled in Supplementary Table S3 (entitled “Summary of Experimental Mouse Cohorts”) in the Supplementary Materials. Behavioral tests were performed during the light circle (09:00 to 18:00). The mice were randomly allocated to a group and transferred to the testing room at least 1 h before the test. The background noise and brightness in the laboratory were maintained at ± 60 dB and below 20 lx, respectively. The personnel preforming the test was unaware of the conditions of the experimental group.

#### Open field test (OFT)

Place the mice (8–12 weeks old) in the center of an open field (100 × 100 × 50 cm) and allow them to move freely for 10 min using a video tracking system. The behavioral parameters were analyzed using SMART software (v3.0, Panlab, Spain) to evaluate murine neurobehavioral profiles. Total distance reflects basal locomotor activity and exploratory drive, with reduced values indicating hypoactivity or depressive-like states. Time in the central zone inversely correlates with anxiety levels, as rodents exhibit thigmotaxis (preferential wall exploration) under anxiety. Central zone entries quantify exploratory motivation, with decreased frequency suggesting behavioral inhibition characteristic of anxiety disorders.

#### Elevated plus maze (EPM)

Mice aged 8–12 weeks were placed in an EPM apparatus, which is being recorded by a camera, for 5 min. The EPM apparatus consists of two open arms (50 × 10 cm), two closed arms (50 × 10 cm and 30 cm wall height), and a central platform (5 × 5 cm), positioned 50 cm above the ground. Place each mouse on the central platform, facing one of the open arms, and allow it to move freely. Locomotion activity is measured using SMART 3.0 software. The following ethological indices were quantified: total distance, reflecting basal motor activity to control for confounding locomotor impairments; time in open arm, inversely correlating with anxiety levels due to innate aversion to open/exposed spaces; entries in open arm, indicating risk exploration propensity. These indices collectively operationalize anxiety-like phenotypes through ethologically validated conflict between innate curiosity and predator-avoidance instincts.

#### Tail suspension test (TST)

Behavioral despair was quantified using the tail suspension test paradigm validated for depression-like phenotype assessment. Individual 8–12 weeks old mice were subjected to the tail suspension paradigm under controlled ambient conditions. Position the tape approximately 1 cm from the tip of the mouse’s tail, and suspend the mouse individually on a platform 50 cm above the ground. Film the animal for 6 min, and assess the time spent immobile during the last 4 min by an investigator blinded to the experimental group.

#### Resident-juvenile-intruder test (RIT)

The resident mice (8–12 weeks old) were permitted to freely explore within their cages. A novelty and juvenile (4-week-old) mouse whose sex is the same with the resident was placed in the cage for 10 min as an intruder will be changed in each test. The duration of direct contact (sniffing, chasing, grooming) reflecting social ability was analyzed by an observer who was not acquainted with the experimental group.

#### Separation-induced vocalization (SIV)

Neonatal P9 mice were brought from their home cage where the dam was the only adult in the cage to a square transparent container (32 cm×20 cm×30 cm) in the testing room. An Ultramic384K BLE ultrasound microphone (Dodotronic, France) that is sensitive to frequencies from 0 to 192 kHz was used to record the sounds of pups for 10 min. Microphone over the pups at a fixed distance of 10 cm. Sounds were acquired by SeaWave 2.0 (Avisoft Bioacoustics, Germany) and analyzed by Deep Squeak 3.0 that can automatically recognize several different separation-induced ultrasonic vocalization (USV) types reflecting sociability of pups and calculate quantitative parameters including the total number from 20 to 120 kHz and mean duration and frequency of the calls.

#### Maternal care behavior

First-delivery pregnant mice were feed separately and provided cotton fibers to build nests before parturition. The picture of newly built nests was photted on the parturition day (P0). Nest quality was measured by an independent observer using the previously described scoring system as follows: grade 3, resembling a deep depression surrounded by high banks; grade 2, a moderately high banked depression; grade 1, flat with low banks, yet still discrete; grade 0, no depression in the substrate and no banks. The number of total pups and pups that stomach filled with milk or body poorly cleaned was recorded at P0. Besides, the number of survival pups was recorded from P0 to P6. Dams were sacrificed 90 min after specific maternal behaviors (e.g., sustained licking/grooming, pup retrieval, nursing or nest construction) at P7 to assess the number of FOS-positive cells. Litters were immediately fostered to synchronized lactating dams, ensuring survival post-sacrifice. Each pup, excluding those involved in cross-fostering, was weighed at P7, P14, and P21.

#### Pup retrieval test

As described previously [21], pups were separated from dams for 30 min before the test at P0. Then three pups were returned to the dam cage and placed in each of the three corners that opposite to the nest. Using a camera on the top of the home cage to record the latency period of the dam first sniff the pups and return each pup to the nest, reflecting the initiation speed of maternal behavior and the persistence and execution efficiency of maternal behavior, respectively.

### Statistics and reproducibility

Data analyses were conducted by a researcher who is unaware of the experimental design. All statistical analyses and graphical representation were carried out using GraphPad Prism 9.0. Data were expressed as means ± SEM. The Shapiro-Wilk test was employed for normality assessment. The Levene test was utilized for homogeneity of variance. Data sets fulfilled both criteria were analyzed via either Two-tailed unpaired t-test or Two-way ANOVA followed by Bonferroni’s test. Otherwise, nonparametric statistical analyses were conducted using the Mann-Whitney U test or Welch’s t-test. *P* < 0.05 was considered statistically significant.

## Results

### Pygb-KI mice exhibit glycogen depletion in astrocytes of both sexes

Astrocyte-specific Pygb-KI mice were generated as depicted in Fig. 1a. All mice of this strain underwent genotype testing and subsequently categorized based on the results (Fig. 1b). The efficiency of Pygb overexpression was confirmed through western blot (Fig. 1c-d), two-way ANOVA showed a main effect of genotype (*n* = 4, F_1,12_=19.36, *p* = 0.0009) with no significant effect of sex or interaction. *Post hoc* analysis with Bonferroni corrections further revealed that the protein level of Pygb was increased in both male and female Pygb-KI mice (WT-M vs. KI-M, *n* = 4, *p* = 0.0343; WT-F vs. KI-F, *n* = 4, *p* = 0.0095). The percentage of Pygb co-stained with GFAP was increased in Pygb-KI mice compared with WT mice, with no effect in sex or interaction (Fig. 1e-f) (Two-way ANOVA, *n* = 4, F_1,28_=28.75, *p* < 0.0001). We observed increased Pygb expression in astrocytes of homozygous Pygb-KI mice compared to WT mice in both sexes (Bonferroni corrections, WT-M vs. KI-M, *n* = 4, *p* = 0.0047; WT-F vs. KI-F, *n* = 4, *p* = 0.0004). Additionally, we assessed brain glycogen content using a glycogen assay kit, through which we discovered reduced glycogen storage in the brain of homozygous Pygb-KI mice compared with WT mice, but no effect in sex or interaction (Fig. 1g) (Two-way ANOVA, *n* = 5, F_1,16_=18.04, *p* = 0.0006). *Post hoc* analysis with Bonferroni corrections further revealed that reduced glycogen storage in both sexes (WT-M vs. KI-M, *n* = 5, *p* = 0.0072; WT-F vs. KI-F, *n* = 5, *p* = 0.0389). Our findings suggest that excessive glycogenolytic capacity leads to deficiency in brain glycogen of Pygb-KI mice regardless of sexes.

### Glycogen depletion impairs the capacity of glycolysis in astrocytes

**Fig. 2:**
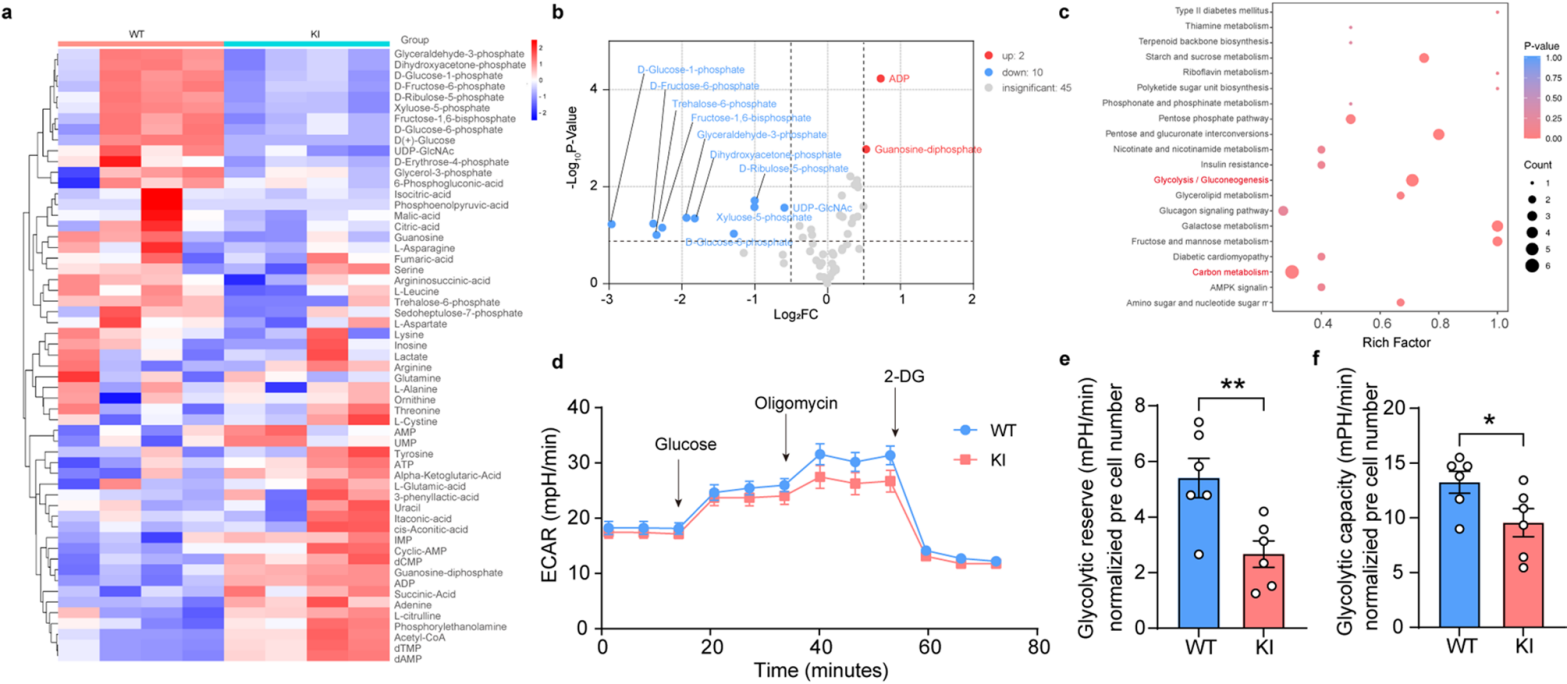
Decreased glycolysis in Pygb-KI mice. (**a**) Cluster analyses of differentially expressed metabolites among WT and Pygb-KI mice. Metabolite levels are shown as colored boxes; The color scale represents row-wise z-score normalized values, with red indicating upregulation and blue denoting downregulation. (**b**) Volcano plot showing detected differential metabolites in Pygb-KI mice compared with WT. Differential metabolites are designated in red (upregulation [up]) or blue (downregulation [down]) and are defined as having an FDR of less than 0.05 FC (fold change). (**c**) KEGG enrichment analysis showing significant alteration of pathways in Pygb-KI mice compared with WT. (**d-e**) ECAR assay cultured WT and Pygb-KI astrocytes. The data are denoted as the mean ± SEM. *P < 0.05, **P < 0.01

In light of the contribution of glycogen on energy metabolism [22], we performed targeting metabolome profiling analysis to investigate alterations in brain metabolites. Heat map and volcano plot disclosed that Pygb overexpression altered metabolic levels, including the reduction of D-glucose-6-phosphate, D-glucose-1-phosphate, D-fructose-6-phosphate, fructose-1,6-bisphosphate, D-ribulose-5-phosphate, xyluose-5-phosphate, glyceraldehyde-3-phosphate, dihydroxyacetone-phosphate, UDP-GlcNAc, trehalose-6-phosphate, and the enhancement of ADP and Guanosine-diphosphate as shown in Fig. 2a-b. KEGG enrichment suggested that the changes of these metabolites were mostly located in the glycolysis/gluconeogenesis pathway attaching to carbon metabolism (Fig. 2c). qPCR testified that the transcriptional level of hexokinase 2 (HK2) decreased, whereas the transcription level of glucose-6-phosphate dehydrogenase (PGD) increased (Fig. S1) (Two-tailed unpaired t-test, Fig. S1a: *n* = 4, t = 3.225, *p* = 0.0180; Fig. S1b: *n* = 4, t = 2.457, *p* = 0.0493). Other key enzymes involved in the glycolysis pathway did not show substantial changes (Fig. S1) (Two-tailed unpaired t-test, *n* = 4, *p* > 0.05). Afterwards, we performed ECAR (External Circulation Anaerobic Reactor) analysis to identify the glycolytic activity in astrocytes (Fig. 2d). Our data revealed that both glycolytic reserve and glycolytic capacity were attenuated in the primary cultured astrocytes of Pygb-KI group (Fig. 2e-f) (Two-tailed unpaired t-test, Fig. 2e: *n* = 6, t = 3.218, *p* = 0.0092; Fig. 2f: *n* = 6, t = 2.276, *p* = 0.0461). These abovementioned results indicate that glycogen depletion in astrocytes causes severe disruptions in energy metabolic patterns.

### Sholl analysis prompts the decreases in area, branching, and length in astrocytes and loss of mature dendritic spines in female but not male mice

**Fig. 3:**
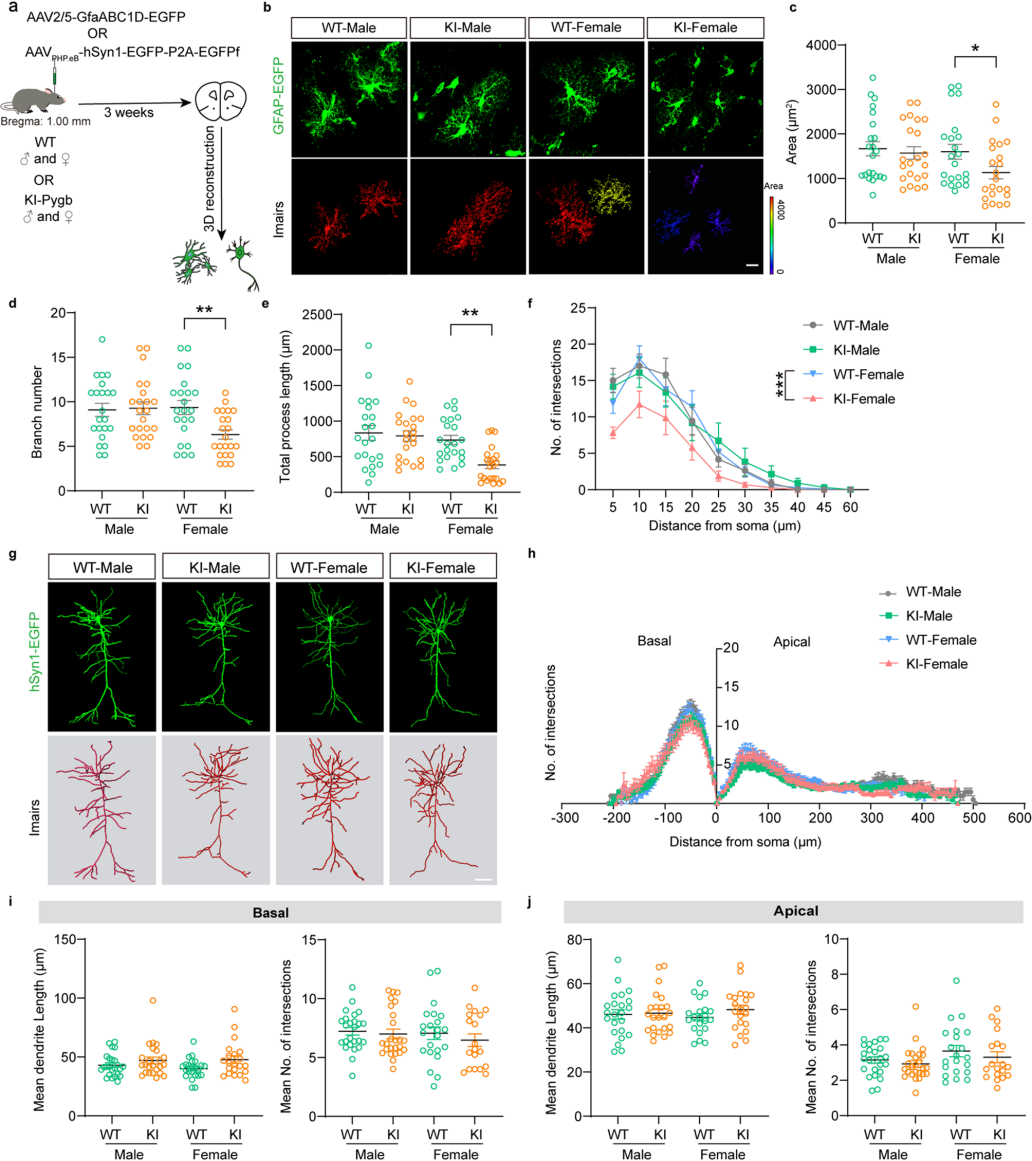
Glycogen deficiency of astrocytes contributes to female-specific decreases in area, branching, and length in astrocytes. (**a**) Schematic of the viral strategy. (**b-f**) Representative imaging (**b**) and quantification (the area (**c**), branch number (**d**), total processes (**e**) and sholl analysis (**f**)) of astrocytes labeled with GFAP-EGFP from the prefrontal cortex of WT and Pygb-KI mice in different sex (scale bar, 10 μm). (**g-j**) Representative imaging (**g**) and quantification (sholl analysis in basal and apical dendrites (**h-j**)) of the pyramidal neurons labeled EGFP from the WT and Pygb-KI mice in different sex (scale bar, 50 μm). The data are denoted as the mean ± SEM. *P < 0.05, **P < 0.01, ***P < 0.001

**Fig. 4:**
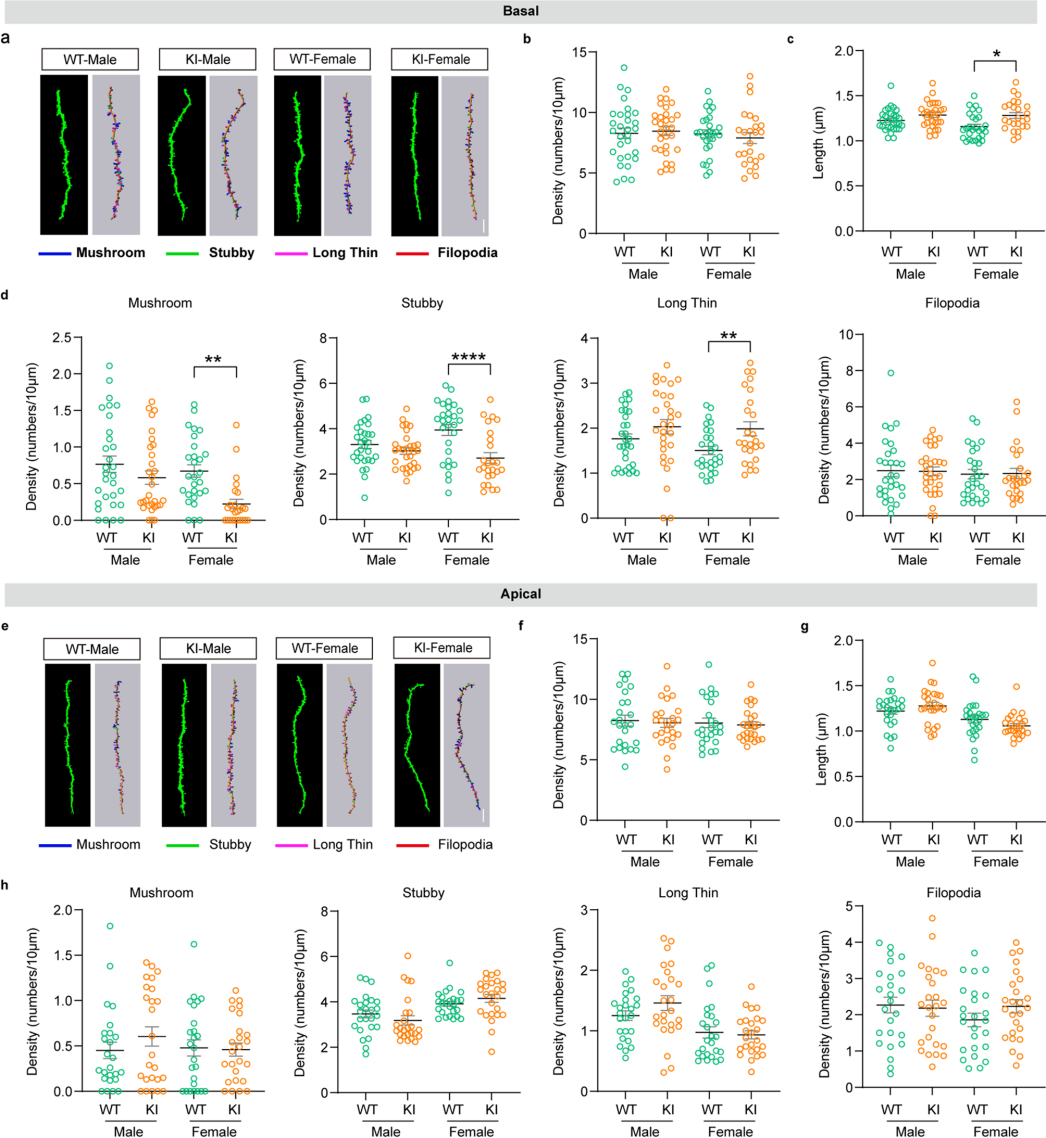
Glycogen deficiency of astrocytes induces basal dendritic spine remodeling of pyramidal neurons. (**a-c**) Representative imaging (**a**) and quantification (the density (**b**), the mean length (**c**)) of basal dendrites of pyramidal neurons obtained from mice expressing EGFP in both WT and Pygb-KI mice in different sex (scale bar, 5 μm). (**d**) Summary of the density of mushroom, stubby, long-thin, and filopodia shaped spines on basal dendrites of pyramidal neurons from mice of the above 4 groups. (**e-g**) Representative imaging (**e**) and quantification (the density **f**), the mean length (**g**)) of apical dendrites of pyramidal neurons obtained from mice expressing EGFP in both WT and Pygb-KI mice in different sex (scale bar, 5 μm). (**h**) Summary of the density of mushroom, stubby, long-thin, and filopodia shaped spines on apical dendrites of pyramidal neurons from mice of the above 4 groups. The data are denoted as the mean ± SEM. *P < 0.05, **P < 0.01, ***P < 0.001

Previous studies have demonstrated the huge impacts of metabolic patterns in astrocytes on structural plasticity themselves and on neurons [23]. Whether glycogen depletion leads to morphological changes in astrocytes and adjacent neurons is still unclear. Here, we labeled these cell types by using targeting virus tools and conducted 3D reconstruction via Imaris software. Specifically, AAV2/5-GfaABC1D-EGFP virus was injected into the prefrontal cortex, and AAV_PHP.eB_-hSyn1-EGFP-P2A-EGFPf virus was injected into retro-orbital sinus to visualize the morphology of astrocytes and neurons, respectively (Fig. 3a). We examined the total area of astrocytes in animals injected by AAV2/5-GfaABC1D-EGFP virus, and two-way ANOVA showed a main effect of genotype (Fig. 3b-c) (*n* = 22 astrocytes from 3 mice, F_1,84_=4.503, *p* = 0.0368) but no significant effect of sex or interaction were found. Following Bonferroni’s corrections, exclusively among Pygb-KI female mice, there was a significant decrease in the total area of astrocytes compared with WT females (*n* = 22 astrocytes from 3 mice, *p* = 0.0264). Two-way ANOVA analysis of branch number in astrocytes of these animals revealed main effects of genotype and interaction (Fig. 3d) (*n* = 22 astrocytes from 3 mice, genotype: F_1,84_=4.219, *p* = 0.0431; interaction: F_1, 84_=5.358, *p* = 0.0231). Again, no significant effect of sex was found, but Bonferroni corrections revealed a decrease in the branch number in the Pygb-KI female mice (*n* = 22 astrocytes from 3 mice, *p* = 0.0054), which was not observed in Pygb-KI males. Two-way ANOVA on the process length of astrocytes showed the main effects of genotype and interaction (Fig. 3e) (*n* = 22 astrocytes from 3 mice, genotype: F_1,84_=6.806, *p* = 0.0108; interaction: F_1,84_=4.152, *p* = 0.0447). *Post hoc* analysis with Bonferroni corrections further revealed a decrease in the length of astrocytes in the KI-female mice (*n* = 22 astrocytes from 3 mice, *p* = 0.0089) compared with WT females. Furthermore, the number of intersections, we observed significantly decline in KI-Female mice. (Fig. 3f) (*n* = 22 astrocytes from 3 mice, F_1,840_=9.940, *p* < 0.0001, Bonferroni corrections, *p* = 0.0002). Sholl analysis of neurons revealed no significant differences in the complexity of apical and basal dendrites across the groups (Fig. 3g-j) (*n* = 19–25 pyramidal neurons from 3 mice, Two-way ANOVA, *p* > 0.05). As mature dendritic spines serve as structural and functional hubs for stable synaptic transmission, long-term potentiation (LTP), etc [24, 25], we also observed changes in dendritic spines and found that there were no significant differences in the total spine density (Fig. 4a-b) (*n* = 25–30 pyramidal neurons from 3 mice, Two-way ANOVA, *p* > 0.05). But considering the lengths in total spines of basal dendrites, Two-way ANOVA showed main effect of genotype (Fig. 4c) (*n* = 25–30 pyramidal neurons from 3 mice, F_1,109_=11.76, *p* = 0.0009) while no effect of sex or interaction was found. *Post hoc* analysis with Bonferroni corrections further revealed that, Pygb-KI led to the lengths in total spines of basal dendrites in female mice increased (*n* = 25–30 pyramidal neurons from 3 mice, *p* = 0.017). Additionally, we performed high-resolution analysis to classify the subtypes of spines, including mushroom, stubby, long-thin and filopodia (Fig. 4d). Two-way ANOVA showed the main effects of genotype and interaction in mushroom (*n* = 25–30 pyramidal neurons from 3 mice, genotype: F_1,109_=11.59, *p* = 0.0009; interaction: F_1,109_=5.927, *p* = 0.0165) and stubby spines (*n* = 25–30 pyramidal neurons from 3 mice; genotype: F_1,106_=22.42, *p* < 0.0001; interaction: F_1,106_=10.56, *p* = 0.0016), and the main effect of genotype in long-thin spines (*n* = 17–30 pyramidal neurons from 3 mice, F_1,98_=10.64, *p* = 0.0015), while none effect was found in filopodia spines (*n* = 25–30 pyramidal neurons from 3 mice, *p* > 0.05) in basal dendrites of neurons. Further analysis of the main composition method by Bonferroni corrections showed that, the number of mushroom (*n* = 25–30 pyramidal neurons from 3 mice, *p* = 0.0025) and stubby (*n* = 25–30 pyramidal neurons from 3 mice, *p* < 0.0001) spines were decreased, long-thin spines were increased (*n* = 17–30 pyramidal neurons from 3 mice, *p* = 0.0064) in basal dendrites of neurons in the KI-female group. Distinct from basal dendrites, the total density, lengths as well as the number of subtype spines of apical dendrites were not changed among these groups (Two-way ANOVA, *p* > 0.05, Fig. 4e-h). These data indicate that glycogen depletion in astrocytes may contribute to the female-specific decreases in area, branching, and length in astrocytes, as well as loss of mature dendritic spines in pyramidal neurons.

### Pygb-KI female mice present anxiety-like behaviors in adulthood, regardless of the virgin or reproductive periods

**Fig. 5:**
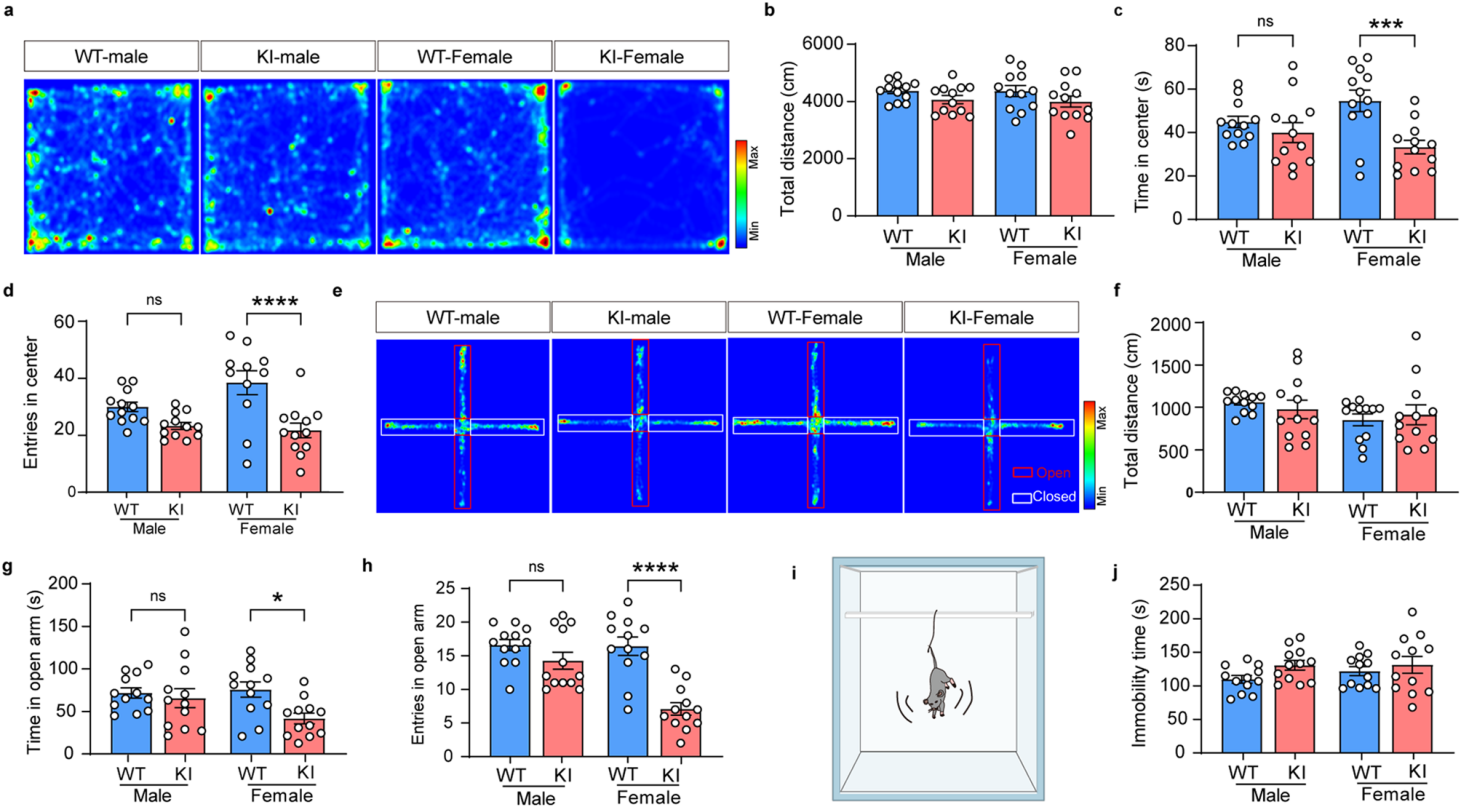
Glycogen deficiency of astrocytes induces anxiety-like behaviors in Pygb-KI female, instead of male mice. (**a-d**) Representative heat map (**a**) and quantitation (total distance (**b**), time in center (**c**), entries in center (**d**)) in the paradigm of OFT for WT and Pygb-KI mice in different sex. (**e-h**) Representative heat map (**e**) and quantitation (total distance (**f**), time in open arm (**g**), entries in open arm (**h**)) in the paradigm of EPM for WT and Pygb-KI mice in different sex. (**i-j**) Schematic diagram of TST (**i**) and total immobility time (**j**) in TST. The data are denoted as the mean ± SEM. *P < 0.05, ***P < 0.001, ****P < 0.0001

Considering the sex-dependent influences of glycogen depletion on brain plasticity, we performed behavioral tests to evaluate its impacts on emotional states of mice in both sexes. During the virgin period in adulthood, there were no significant differences in total distances in the OFT (Fig. 5a-b) (*n* = 12, Two-way ANOVA, *p* > 0.05), demonstrating the identical baseline of locomotor activity in WT and KI mice. Whereas, it was observed main effects of genotype and interaction in the time spent in the center zone with Two-way ANOVA (Fig. 5c) (*n* = 12, genotype: F_1,44_=10.96, *p* = 0.0019; interaction: F_1,44_=4.269, *p* = 0.0447). KI-female spent less time in the center zone compared with WT-Female (*n* = 12, Bonferroni corrections, *p* = 0.009). For the number of entries into the center zone, only the effect of genotype was found and revealed a reduced number of entries into the center zone in KI-females compared with WT-females (Fig. 5d) (*n* = 12, Two-way ANOVA, F_1,44_=20.19, *p* < 0.0001; Bonferroni corrections, *p* < 0.0001). In the EPM, we also observed no marked changes in total distances (Fig. 5e-f) (*n* = 12, Two-way ANOVA, *p* > 0.05), while the time spent (*n* = 12, *p* = 0.017) and number of entries into the open arm (*n* = 12, *p* < 0.0001) were significantly decreased in the KI-female group compared with WT-Female. It was also observed a genotype of effect in the time spent (Fig. 5g) (*n* = 12, F_1,44_=5.818, *p* = 0.0201) and both genotype and interaction effects in the number of entries into the open arm with Two-way ANOVA (Fig. 5h) (*n* = 12, genotype: F_1,44_=27.10, *p* < 0.0001; interaction: F_1,44_=7.756, *p* = 0.0032). In the TST, immobility time did not show significant changes among groups (Fig. 5i-j) (*n* = 12, Two-way ANOVA, *p* > 0.05). These findings manifest that glycogen depletion in astrocytes distinctively induces anxiety-like, but not depressive-like behaviors in female mice.

**Fig. 6:**
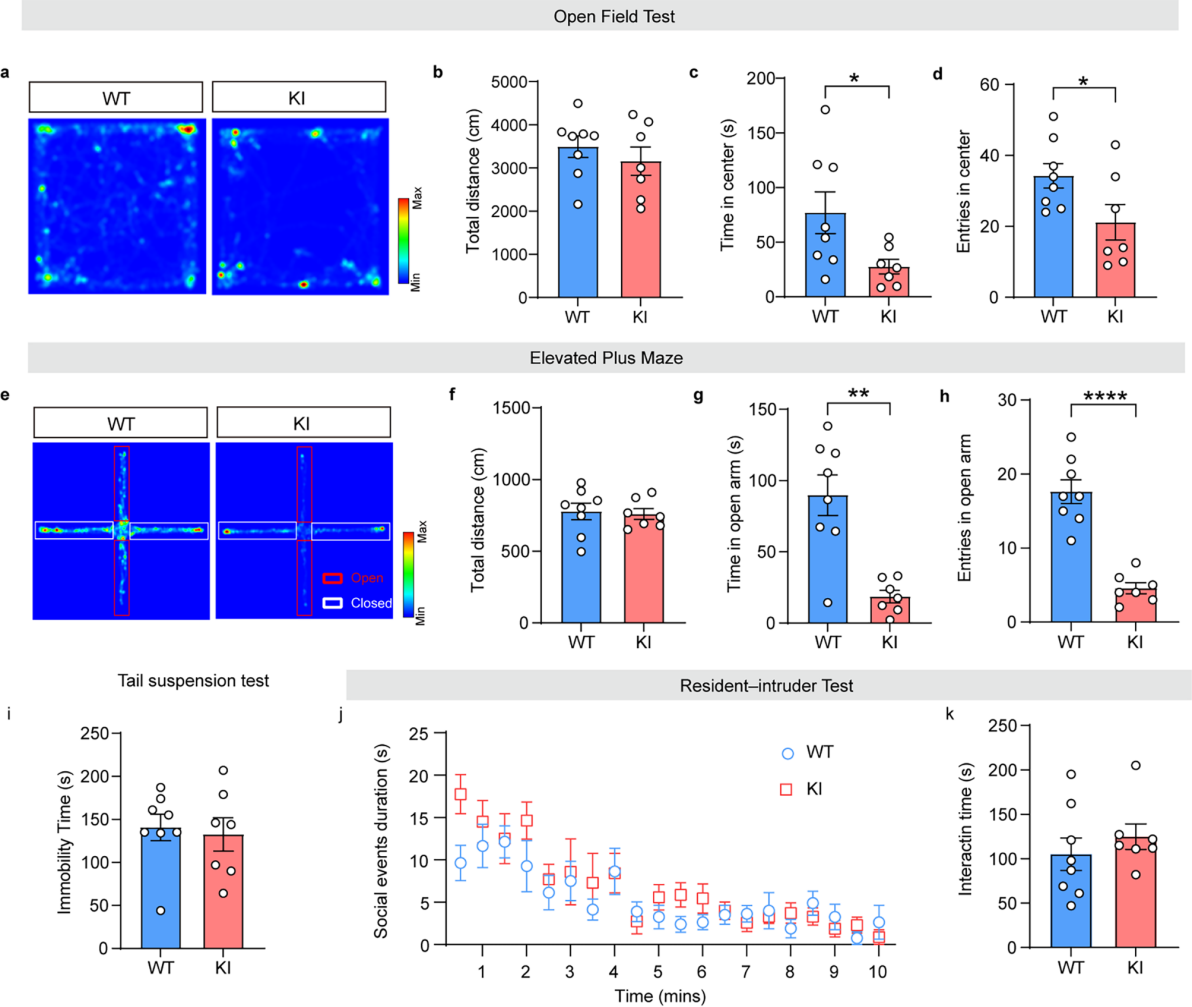
Glycogen deficiency of astrocytes results in anxiety-like behaviors during lactation period. (**a-d**) Representative heat map (**a**) and quantitation (total distance (**b**), time in center (**c**), entries in center (**d**)) in the paradigm of OFT for WT and Pygb-KI lactating females. (**e-h**) Representative heat map (**e**) and quantitation (total distance (**f**), time in open arm (**g**), entries in open arm (**h**)) in the paradigm of EPM for WT and Pygb-KI lactating females. (**i**) Total immobility time in TST. (**j-k**) The behavioral quantitation of resident-intruder test of WT and Pygb-KI lactating females. The data are denoted as the mean ± SEM. *P < 0.05, **P < 0.01, ***P < 0.001

Subsequently, we examined whether glycogen depletion affected the above-mentioned behavioral responses in the reproductive period. Dams at 1–3 days after delivery received behavioral tests. OFT showed that the total distances were statistically the same between WT and KI groups (Fig. 6a-b) (*n* = 7–8, Mann–Whitney U test, *p* > 0.05), while the time spent and number of entries into the center zone were remarkably decreased in KI lactating mice (Fig. 6c, *n* = 7–8, Mann–Whitney U test, U = 10, *p* = 0.0401; Fig. 6d, *n* = 7–8, Two-tailed unpaired t-test, t = 2.212, *p* = 0.0455). In the EPM, there were also no significant differences in the total distances (Fig. 6e-f) (*n* = 7–8, Two-tailed unpaired t-test, *p* > 0.05), while the time spent and number of entries into the open arm were notably diminished (Fig. 6g, *n* = 7–8, Mann–Whitney U test, U = 4, *p* = 0.0037; Fig. 6h, *n* = 7–8, Two-tailed unpaired t-test, t = 7.025, *p* < 0.0001). The immobility time in the TST and the social events duration in the RIT were not obviously changed, suggesting there were non-existences in depressive-like behaviors and social deficits (Fig. 6i-k, *n* = 7–8, Welch’s t-test, *p* > 0.05). These data reveal that glycogen depletion also causes anxiety-like behaviors during the lactation period.

### Glycogen depletion induces maternal care obstacles in lactating mice

**Fig. 7:**
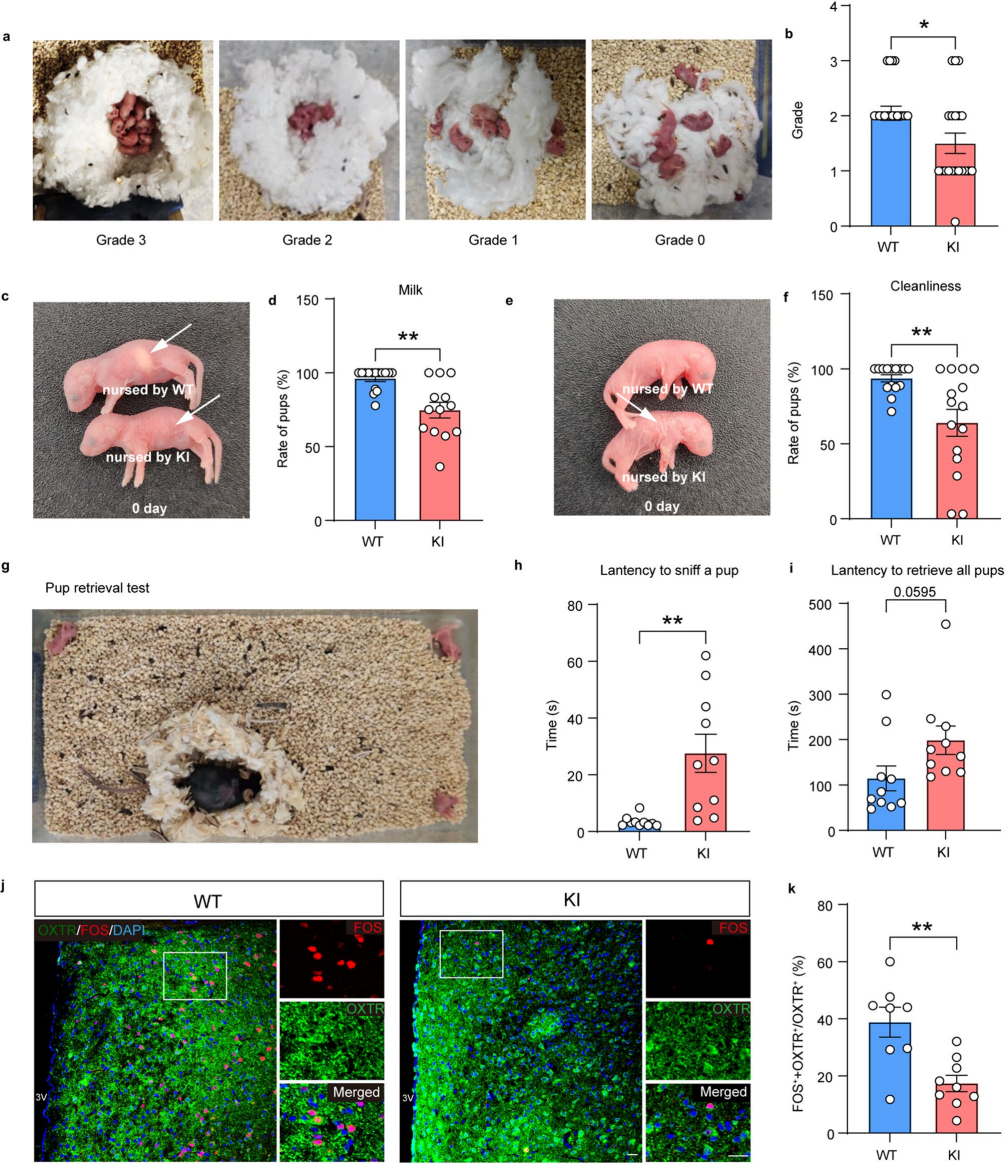
Glycogen depletion induces maternal care obstacles in lactating female mice. (**a-b**) Representative images of nests in different grade (**a**) and the nest quality scores (**b**). (**c**) Representative image of pups nursed by WT had milk in their digestive tract (arrow), whereas pups nursed by Pygb-KI dams did not. (**d**) Ratio of pups with milk in the digestive tract. (**e**) Representative image of pups nursed by WT were cleaned (arrow), whereas pups nursed by Pygb-KI dams did not. (**f**) Ratio of cleaned pups. (**g-i**) Representative image (**g**) and quantitation (latency to sniff a pup for the first time (**h**), latency to retrieve all pups (**i**)) in the retrieval test. (**j-k**) Representative images (**j**) and quantification (**k**) of the co-expression of OXTR (green) and FOS (red) in the MPOA of first-delivery pregnant WT and Pygb-KI mice. Scale bars = 50 μm. The data are denoted as the mean ± SEM. *P < 0.05, **P < 0.01

There is a close correlation between negative emotions and maternal care deficits in females [26]. Thus, we conducted a series of behavioral tests to validate the hypothesis that glycogen depletion may cause maternal care obstacles in mice. Our data showed that the quality of nests constructed by KI-dams was inferior to those built by WT-dams (Fig. 7a-b, *n* = 20–21, Welch’s t-test, t = 2.431, *p* = 0.0205). Compared to WT-dams, KI-dams exhibited a lower proportion of offspring with milk in their digestive tract that also receive adequate cleanliness (Fig. 7c-f) (Fig. 7d, *n* = 13–15, Welch’s t-test, t = 3.775, *p* = 0.0018; Fig. 7f, *n* = 15, Welch’s t-test, t = 3.200, *p* = 0.0056). Afterwards, we monitored dam’s responses in the pup retrieval test. KI-dams exhibited significantly delayed sniffing and trend of delayed retrieving behaviors towards the pups when compared with WT-dams (Fig. 7g-i) (Fig. 7h, *n* = 10, Welch’s t-test, t = 3.584, *p* = 0.0057; Fig. 7i, *n* = 10, Welch’s t-test, t = 2.014, *p* = 0.0595). In view of the activation of oxytocin receptor positive neurons in the MPOA of the rostral hypothalamus when a mouse engages in caregiving behaviors [27], we quantified the number of FOS-positive cells in the MPOA of female mice while they were nurturing their offspring and found that compared with WT-dams, the KI group exhibited a significant reduction in the number of FOS^+^/OXTR^+^ cells (Fig. 7j-k) (*n* = 4, Two-tailed unpaired t-test, t = 3.726, *p* = 0.0020). These results indicate that glycogen depletion disrupted neural mechanisms responsible for maternal behaviors.

### Reduced survival rate and ultrasonic vocalization calls in pups reared by Pygb-KI dams

**Fig. 8:**
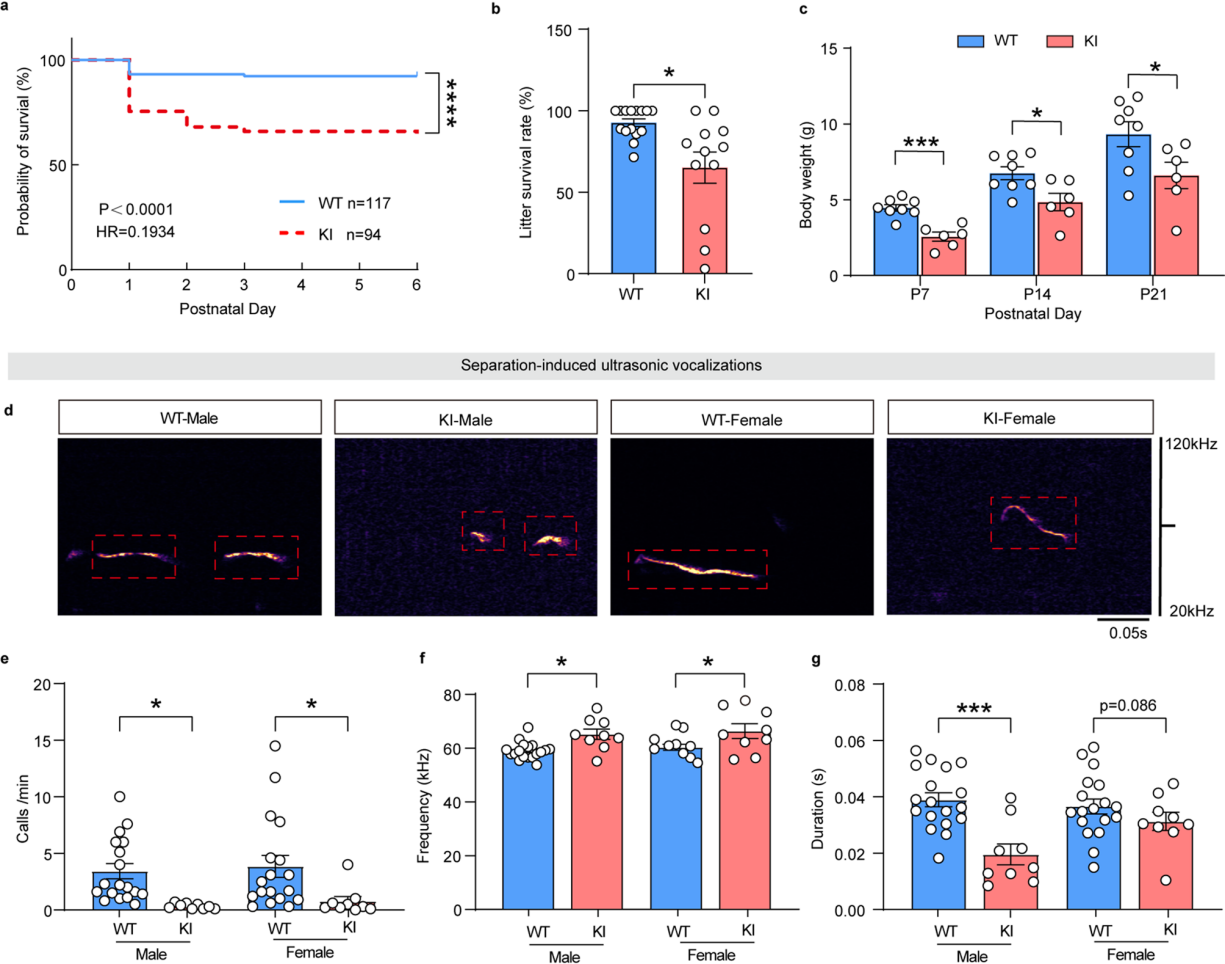
Survival rate and sociability deficits in pups raised by KI-dams. (**a**) The survival curve of pups raised by WT or Pygb-KI dams. (**b**) The rate of survival pups per litters at P6 raised by WT or Pygb-KI dams. (**c**) Weight of pups raised by WT or Pygb-KI dams. **d-g** Representative USV recordings (**d**) and quantitation (numbers (**e**), duration (**f**) and frequency (**g**) of the calls) in the paradigm of separation-induced ultrasonic vocalizations for WT or Pygb-KI pups at P9. The data are denoted as the mean ± SEM. *P < 0.05, **P < 0.01, ***P < 0.001

To confirm whether glycogen depletion impacts the survival and behavioral phenotypes of pups, we recorded the development of offspring mice during the early stage after birth. The survival rate and body weight of pups nurtured by KI-dams were dramatically lower than those nurtured by WT-dams (Fig. 8a-c) (Fig. 8a, *n* = 117 for WT-dams and *n* = 94 for KI-dams, Log-rank (Mantel-Cox) test, Chi square = 24.57, *p* < 0.0001; Fig. 8b, *n* = 15 for WT-dams and *n* = 12 for KI-dams, Welch’s t-test, t = 2.798, *p* = 0.0157; Fig. 8c, *n* = 8 for the WT group and *n* = 6 for the Pygb-KI group, Two-tailed unpaired t-test, P7: t = 5.396, *p* = 0.0002; P14: t = 2.725, *p* = 0.0184: P21, t = 2.229, *p* = 0.0457). Subsequently, we recorded separation-induced ultrasonic vocalization calls to assess the communicative competence (Fig. 8d). The results declared that the call rates of pups in both sexes were reduced (Fig. 8e) (*n* = 18 for the WT group and *n* = 9 for the Pygb-KI group, Two-way ANOVA, genotype: F_1,50_=13.06, *p* = 0.0007, Bonferroni corrections, male: *p* = 0.0271; female: *p* = 0.0277), while the frequency of calls was enhanced in pups raised by KI-dams when compared with those raised by WT-dams (Fig. 8f) (*n* = 18 for the WT group and *n* = 9 for the Pygb-KI group, Two-way ANOVA, genotype: F_1,50_=16.70, *p* = 0.0002, Bonferroni’s multiple comparisons test, male: *p* = 0.0106; female: *p* = 0.0123). The duration of calls was overall decreased, though there was no statistical difference in the female group (Fig. 8g) (*n* = 18 for the WT group and *n* = 9 for the Pygb-KI group, Two-way ANOVA, genotype: F_1,50_=20.79, *p* < 0.0001, Bonferroni corrections, male: *p* = 0.0001; female: *p* = 0.0860). Accordingly, we infer that due to maternal care obstacles and emotional distress, glycogen depletion results in reduced survival rate and sociability defects in the offspring mice.

### Alterations of estrogen and glucocorticoid receptors in the Pygb-KI mice at the lactation stage

**Fig. 9:**
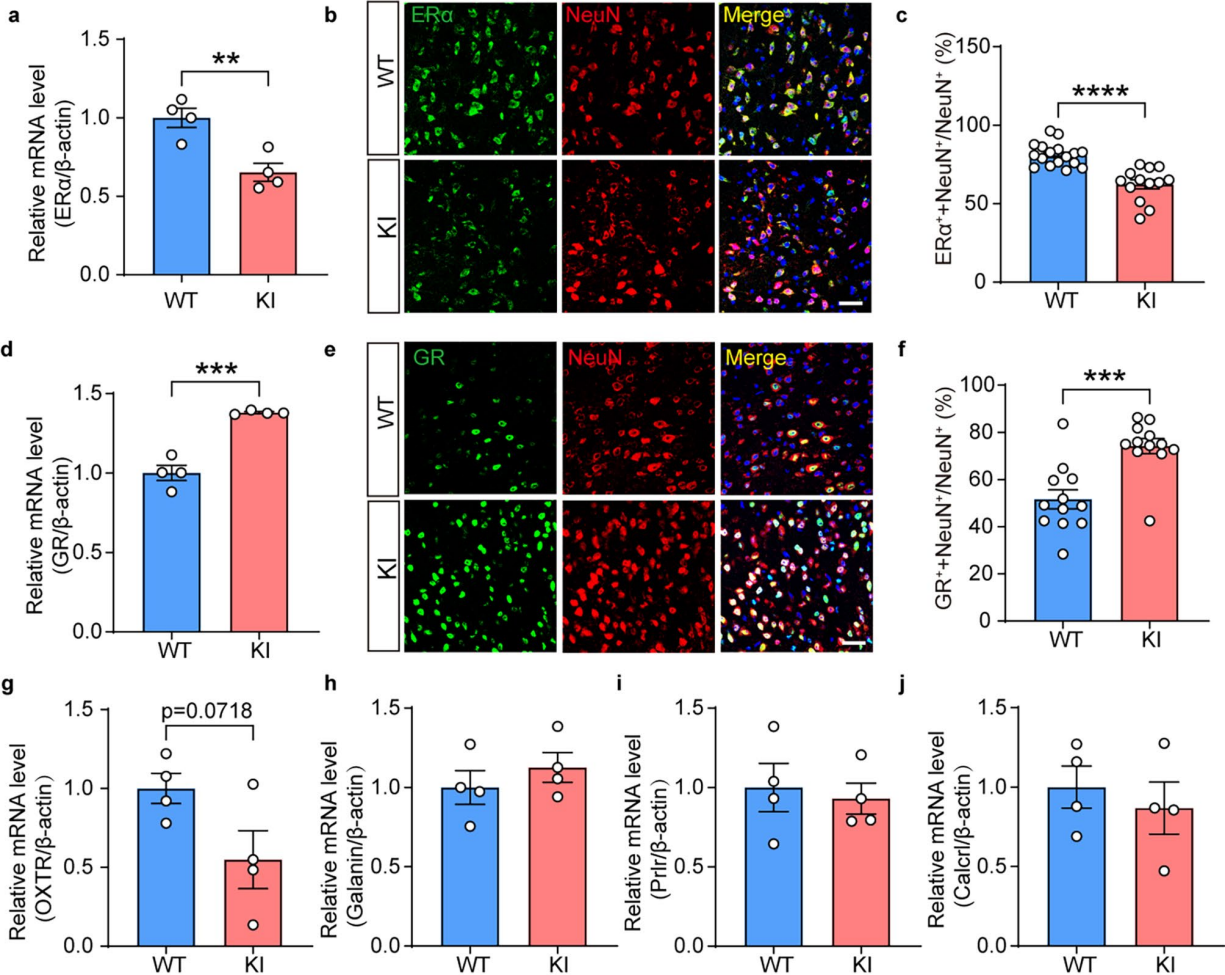
Estrogen receptors and glucocorticoid receptors exhibited opposite tendencies in the Pygb-KI mice at the lactation stage. (**a**) Quantitative real-time PCR analysis of the relative mRNA expression of ERα. **b-c** Representative images (**b**) and quantification (**c**) of the co-expression of ERα (green) and NeuN (red) in the MPOA of WT and Pygb-KI mice. Scale bars = 50 μm. (**d**) Quantitative real-time PCR analysis of the relative mRNA expression of GR. (**e-f**) Representative images (**e**) and quantification (**f**) of the coexpression of GR (green) and NeuN (red) in the MPOA of WT and Pygb-KI mice. Scale bars = 50 μm. (**g-j**). Quantitative real-time PCR analysis of the relative mRNA expression of genes that pregnancy-related hormones. (Two-tailed unpaired t-test). The data are denoted as the mean ± SEM. *P < 0.05, **P < 0.01, ***P < 0.001, ****P < 0.0001 (Two-tailed unpaired t-test (**a**, **c**, **d** and **g-j**), Welch’s t-test (**f**))

Recent studies have demonstrated that modifications of glial and neuronal plasticity during the transition to motherhood were often attributed to pregnancy hormones, such as estradiol, corticosterone, progesterone and oxytocin [28, 29]. In our study, we noted that among neuronal hormonal receptors, the expression of estradiol receptor alpha (ERα) was decreased while glucocorticoid receptor (GR) was increased in neurons, demonstrating aberrant neuronal hormone responses with glycogen depletion (Fig. 9) (Fig. 9a: *n* = 4, Two-tailed unpaired t-test, t = 4.136, *p* = 0.0061; Fig. 9c: *n* = 4, Two-tailed unpaired t-test, t = 5.774, *p* < 0.0001; Fig. 9d: *n* = 4, Two-tailed unpaired t-test, t = 7.970, *p* = 0.0002; Fig. 9f: *n* = 4, Welch’s t-test, t = 4.339, *p* = 0.0003). We did not observe significant changes in other hormonal signals like oxytocin receptor (OXTR), Galanin, prolactin receptor (Prlr) and calcitonin receptor (Calcrl) (Fig. 9g-j *n* = 4, Two-tailed unpaired t-test, *p* > 0.05). These results revealed significant dysregulations in neuronal hormonal signaling pathways characterized by estradiol signaling deficit and stress signaling elevation in the MPOA of Pygb-KI mice at the lactation stage.

## Discussion

In this study, by generating Pygb-KI mice, we proposed that glycogen depletion in astrocytes led to abnormalities in glycolytic patterns regardless of sexes. It induced impairments in the precise morphology of astrocytes and loss of mature dendritic spines in female mice rather than in males. Therefore, KI-females displayed anxiety-like behaviors during adulthood, regardless of virgin or reproductive period. These sex-dimorphic alterations provided clues that glycogenolysis in astrocytes may contribute to mood disorders and maternal care obstacles during the perinatal period, which have become highly prevalent in recent years [30]. Subsequently, we discovered a series of maternal behavioral obstacles, reduced survival rate and sociability of offspring nurtured by Pygb-KI dams, and dysregulations in neuronal hormonal signaling pathways at the lactation stage. These findings highlight the relationship between astrocytic glycogen metabolism and sex differences in brain structural plasticity, as well as sex-dimorphic behavioral phenotypes, emphasizing the need for effective therapeutics to address postpartum anxiety and maternal behavioral deficits in the female population.

In recent years, emotional challenges during the perinatal period have garnered increasing public attention with the escalation of societal pressure and the improvement of focus on mental health, notably postpartum anxiety and depression [31]. It influences up to 15% of mothers and may cause disabling complications of childbearing, including low mood, tearfulness, irritability, even suicide and hurt towards infants [32]. Nevertheless, the understanding regarding the mechanism underlying mental illness related to childbearing remains inadequate, hindering our ability to identify the best therapeutic interventions for ensuring optimal maternal, infant, and family outcomes. Here, we reported a previously unsuspected phenomenon that astrocytic glycogen depletion in females displayed intensive anxiety-like behaviors in both virgin and reproductive periods. This sex-specific phenotype provides evidence that maternal affective disorders might be susceptible to the brain glycogen changes—a biochemical process that determining metabolic homeostasis and neuronal activity in the CNS.

Principally enriched in the terminals of astrocytes, glycogen granules participate in many physiological processes and safeguard the brain against various bioenergetic insults, such as trauma, hypoxia, ischemia/reperfusion, etc. [33, 34]. According to the “astrocyte-neuron lactate shuttle (ANLS)” theory, astrocytic glycogen assumes a dominant role in the coupling of neuronal synaptic activity with glycolytic metabolism [8]. Glycogenolysis and glycolysis are biochemically coupled processes: glycogen breakdown provides glucose-6-phosphate (G6P), the entry substrate for glycolysis. In Pygb-KI mice, glycogen depletion (Fig. 2a-b) directly reduces intracellular G6P pools, limiting glycolytic flux [35]. Here, we proposed that excessive glycogenolysis with glycogen depletion impaired energy metabolic patterns in astrocytes and synaptic plasticity in neurons, supporting the hypothesis of ANLS to some extent. Beyond the metabolic regulation introduced above, recent studies have also disclosed the non-metabolic functions of glycogen in the regulation of cellular homeostasis, such as glycosylation modification, etc [36]. These state-dependent alterations are reported to cause indelible impacts on the structural and functional characteristics in neuronal cells, and play crucial roles in diverse conditions, encompassing several physiological (sleep, exercise, development, etc.) and pathological states (glycogen storage disease, dementia, traumatic brain injury, etc.) [37].

Our group previously elucidated that dysfunction of glycogenolysis is responsible for cerebral ischemia/reperfusion injury, depression, and delayed arousal from isoflurane anesthesia [15, 16, 38]. Thus, we generated Pygb-KI mice to systematically evaluate the sex-dimorphic functions of brain glycogen in the CNS. In brief, after initially observing the phenotypes of anxiety-like behaviors in female rather than male mice, we identified the existence of severe maternal care impediments in the transgenic mice. In our study, glycogen depletion induced incomplete nest building, pup-feeding and -nursing, and exploratory and investigative behaviors towards pups in dams. Considering the association between poor maternal performance and high risks in children’s internalizing and externalizing psychopathology, we proved that pups raised by Pygb-KI dams acquired worse survival rate, physical development, and social interactions in the early stages after birth, highlighting the impacts of maladaptive mother-baby attachment on pups’ outcomes. While our current study focused on maternal phenotypes, several studies highlight sex-specific metabolic-behavioral interactions. Elevated testosterone enhances ventromedial hypothalamic glucose metabolism to fuel aggression, while intermittent fasting increases male mating success via tryptophan-serotonin modulation [39, 40]. What’s more, glycogen-dependent metabolic support may also regulate estrogen-sensitive circuits governing sexual receptivity which is essential for female sexual receptivity. Future studies will systematically delineate and quantify the effects of glycogen metabolism on sex-specific reproductive behaviors, including mating dynamics and aggressive interactions, in both male and female cohorts, guided by our preliminary metabolic findings.

In rodents and mammals, the arrival of newborns triggers a set of adaptive changes in maternal brain plasticity. Several longitudinal studies indicate that, compared to non-mothers, mothers typically exhibit a reduction in gray matter volume immediately postpartum [41]. Moreover, volume decline in specific nuclei has been positively correlated to increased neural activation related to pup-directed caretaking behaviors [42]. Mechanically, the activation of the maternal care circuit is accompanied by structural neural plasticity, involving morphological changes in neurons and glial cells [1]. Astrocytes exhibit highly complex morphological features, with peripheral processes located near the synapses of neurons within the central nervous system-named tripartite synapse, regulating glutamate uptake, ion homeostasis and so on [23]. These intricate processes contribute to the synaptic function of the host, ultimately influencing the excitatory/inhibitory balance in pyramidal neuron networks—a well-established substrate of anxiety pathophysiology [43]. In our study, we analyzed morphological changes in astrocytes, neurons and dendritic spines in the ACC, based on the consideration that ACC is a hub for integrating emotional and social cognitive processing [44, 45], aligning precisely with our observed anxiety-like and maternal care phenotypes. We observed significant reductions in branch number and process length in astrocytes in female mice following glycogen depletion in the ACC, which specifically followed with loss of mushroom spines in basal dendrites. According to literature, the apical dendrites receive feedback inputs, while basal dendrites receive feedforward inputs within the prefrontal cortex [46]. Dendritic spine simplification diminishes synaptic integration capacity [47]. This dual pathology may synergistically result in the abnormal behavioral phenotypes of the mice. Besides, the female specificity could arise from estrogen-dependent astrocyte-neuron crosstalk via mGluR5/ERα co-signaling [48]. Our observed morphological disruptions are likely to uncouple these trophic interactions, disproportionately affecting female neural networks.

The hormonal fluctuations prominently control maternal behaviors in rodents and mammals [1]. Estrogenic augmentation related to astrocyte glycogen content is likely a clear indicator via opposite changes in glycogen synthase (up-regulated) versus glycogen phosphorylase (down-regulated) activity [12], suggesting that postpartum estrogen withdrawal could precipitate aberrant glycogenolytic processes. Beyond that, circadian desynchronization resulting from fragmented maternal sleep during this period disrupts BMAL1 function, which has been shown to regulate the diurnal rhythms of glycogen and its associated metabolic genes and proteins [49]. Notably, Estrogen (ERα/ERβ) and glucocorticoids (GR) exhibit opposing roles in neuronal resilience—ER promotes synaptic plasticity and neuroprotection (e.g., BDNF upregulation) [50, 51], while chronic GR activation under stress induces dendritic atrophy and excitotoxicity [52]. ER/GR imbalance is implicated in mood-related diseases, where estrogen withdrawal and hypercortisolemia synergistically impair neurogenesis [53]. Emerging evidence have suggested that estrogen’s neuroprotective actions are partially mediated through astrocyte-neuron crosstalk involving glycogen metabolism [54]. The convergence of glycogen-dependent energy buffering failure and reproductive stress may explain the observed exacerbation of affective and behavioral dysregulation in female murine models. Thus, we emphasize the involvement of glycogen metabolism in sex differences of both estrogen signaling pathways and the neuroanatomical substrates beyond pregnancy in females. Oxytocin is considered as a core hormone in dominating maternal behaviors via oxytocinergic neurons located in the MPOA, a specific region within the maternal brain circuitry [1]. Quantification of FOS^+^ cells within the MPOA revealed reduced activation in the OXTR^+^ cells in this region among KI-female mice. These findings indicated that peripartum hormones may collectively participate in regulating maternal behaviors in females. Considering the critical regulatory role of estrous cycle dynamics in rodent behavior and neuroplasticity [55], we used females in natural metestrus/diestrus phases to avoid any cycle-driven confounder in this study. Further study is needed to dissect the relationships between estrous cycle and behavioral/morphological phenotypes in female mice.

Efforts may be taken to enhance the quality of this study. Firstly, the underlying mechanism about how astrocytic glycogen depletion triggering the neuronal plasticity and maternal behavioral phenotypes in female rather than male mice needs to be investigated in future studies. Secondly, considering the significant and intricate hormonal fluctuations during pregnancy and the link between GR upregulation and anxiety-like behaviors [56], the definite influence of glycogen metabolism on hormonal responses as well as the quantification of serum stress hormones, namely, corticosterone, still lacks of evaluation. Thirdly, while our data highlight the role of ACC, the broader implications of astrocytic glycogenolysis program for the onset and activation of maternal circuits across limbic and hypothalamic networks need to be further explored.

## Conclusion

In conclusion, we propose that glycogen in astrocytes plays a pivotal role in modulating neuroplasticity and maternal behaviors during pregnancy and the postpartum period. This sex-dependent process illuminates how astrocytic metabolites instigates neural adaptions to shape maternal behaviors in rodents. Targeting astrocytic glycogen metabolism holds promise for supplying effective therapeutics for addressing postpartum anxiety and maternal behavioral deficits in clinical conditions.

### Perspectives and significance

Altogether, our results demonstrate the sex differences of astrocytic glycogen depletion on structural plasticity of astrocytes and synapses, as well as anxiety-like and maternal behaviors, emphasizing the need for effective therapeutics to address postpartum anxiety and maternal behavioral deficits in the female population.

## Electronic supplementary material

Below is the link to the electronic supplementary material.

Supplementary Material 1

## Data Availability

No datasets were generated or analysed during the current study.
