# Supplementary material for: Glycogen depletion in astrocytes induces sex-dimorphic remodeling of astrocytic and synaptic structures with concomitant anxiety-like behaviors and maternal care deficits
Source: Biol Sex Differ. 2025 Jun 11;16:41. doi: 10.1186/s13293-025-00723-6 (PMC12153178; doi:10.1186/s13293-025-00723-6)
Supplement: Supplementary file 1 — Supplementary Material 1 [file 13293_2025_723_MOESM1_ESM.docx]

**Astrocyte glycogenolytic program participates in anxiety-like and maternal behaviors in female mice**

Xiaotong Shi, Yuanyuan Zhu, Zhaoyichun Zhang, Ningcan Ma, Danyi He, You Wu, Ziyi Dai, Xinyan Qin, Yingyi Chen, Youyi Zhao, Haopeng Zhang, Jing Huang, Hui Zhang, Ze Fan

**Supplementary Information**

- Figure S1
- Table S1
- Table S2
- Table S3


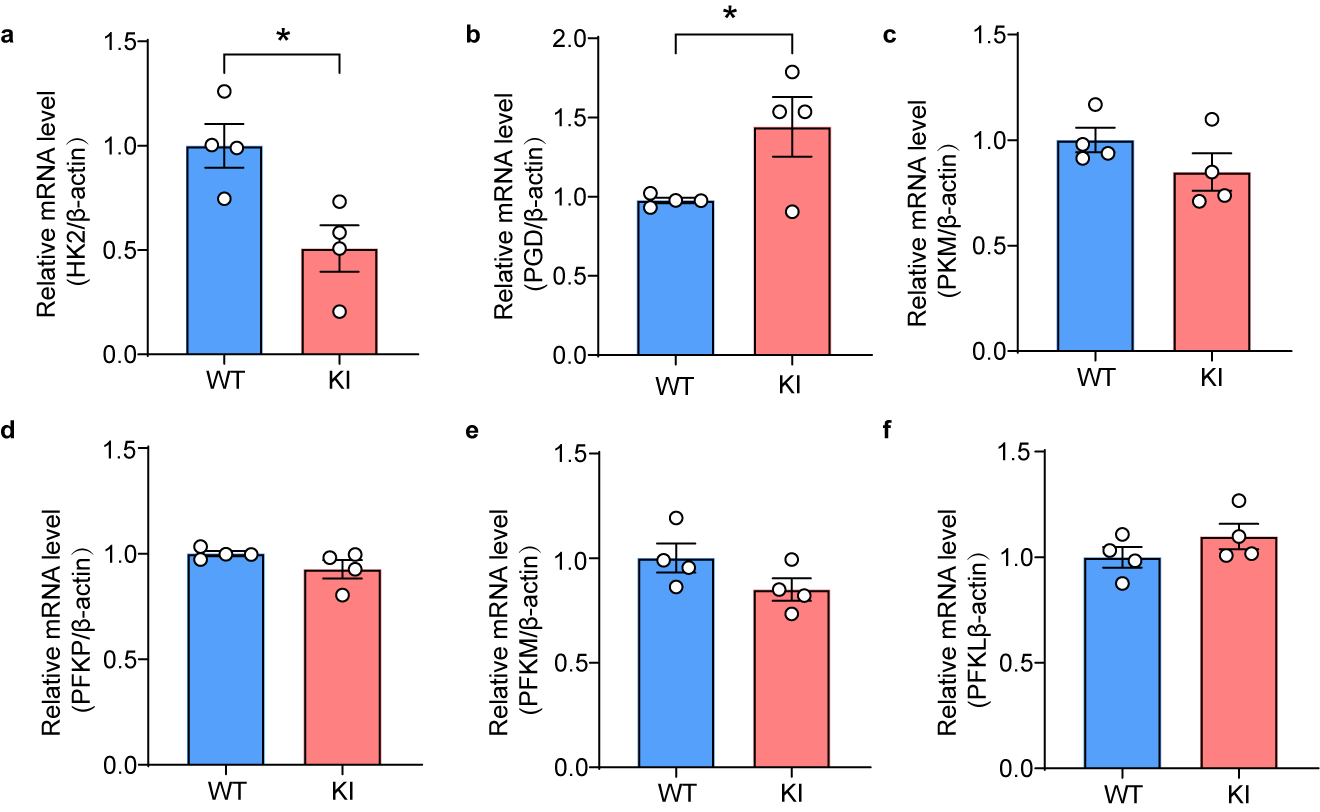
**Figure S1.** Quantitative real-time PCR analysis of the relative mRNA expression of genes that metabolism-related enzymes in the brain of WT and Pygb-KI mice. n=4. The data are denoted as the mean±SEM. *P<0.05 (Two-tailed unpaired t-test).

**Table S1.** Sources of the antibodies used in this study (related to Figure 1,7 and 9)

| Target of antibody | Source | Application | Dilution | Identifier |
| --- | --- | --- | --- | --- |
| PYGB | Proteintech | WB, IF | 1:1000  1:200 | 55380-1-AP |
| GFAP | Sigma-Aldrich | IF | 1:500 | MAB360 |
| c-fos | Synaptic Systems | IF | 1:1000 | 226308 |
| OXTR | Proteintech | IF | 1:50 | 23045-1-AP |
| ERα | Proteintech | IF | 1:200 | 21244-1-AP |
| GR | Proteintech | IF | 1:200 | 24050-1-AP |
| NeuN | Proteintech | IF | 1:500 | 66836-1-Ig |
| β-actin | GeneTex | WB | 1:5000 | GTX11003 |
| Rabbit IgG H&L (HRP) | Proteintech | WB | 1:5000 | SA00001-2 |
| Mouse IgG H&L (HRP) | Proteintech | WB | 1:5000 | SA00001-1 |
| Rabbit IgG H&L  (Fluor 488) | Invitrogen | IF | 1:500 | A21206 |
| Mouse IgG H&L  (Fluor 594) | Invitrogen | IF | 1:500 | A21203 |
| Guinea pig IgG H&L (Fluor 594) | Abcam | IF | 1:500 | ab105118 |

WB: westing blotting; IF: immunofluorescence

**Table S2.** Sequences of the primers used for real-time PCR (related to Figure 1, 9 and Supplementary Figure 1).

| **Gene** | **Sense (5′-3′)** | **Anti-sense (5′-3′)** |
| --- | --- | --- |
| PFKP | GAAACATGAGGCGTTCTGTGT | CCCGGCACATTGTTGGAGA |
| PFKL | GGAGGCGAGAACATCAAGCC | CGGCCTTCCCTCGTAGTGA |
| PFKM | TGTGGTCCGAGTTGGTATCTT | GCACTTCCAATCACTGTGCC |
| HK2 | TGATCGCCTGCTTATTCACGG | AACCGCCTAGAAATCTCCAGA |
| PKM | GCCGCCTGGACATTGACTC | CCATGAGAGAAATTCAGCCGAG |
| PGD | ATGGCCCAAGCTGACATTG | GCACAGACCACAAATCCATGAT |
| OXTR | GGCCGTGTTCCAGGTTCTC | TGCAAGTATTTGACCAGACGAC |
| Galanin | GGCAGCGTTATCCTGCTAGG | CTGTTCAGGGTCCAACCTCT |
| Prlr | GAGAAGGGCAAGTCTGAAGAAC | GGGATGGCATTAGCCGCTC |
| Calcr | GCAACGCTTTCACTTCTGAGA | GTTCCCACTGCATTGTCCACA |
| Gnrh1 | AGCACTGGTCCTATGGGTTG | GGGGTTCTGCCATTTGATCCA |
| GR | AGCTCCCCCTGGTAGAGAC | GGTGAAGACGCAGAAACCTTG |
| Esr1 | CCTCCCGCCTTCTACAGGT | CACACGGCACAGTAGCGAG |
| β-actin | GTCCCTCACCCTCCCAAAAG | GCTgCCTCAACACCTCAACCC |

**Table 3.** Summary of Experimental Mouse Cohorts

| **Data** | **Test** | **Groups** | **Observation** | **Sample size (n)** | **Age** |
| --- | --- | --- | --- | --- | --- |
| Figure1d | WB | C57♂, Pygb-KI♂, C57♀ and Pygb-KI♀ | mouse | 4 | 8-12 weeks |
| Figure1f | IF | C57♂, Pygb-KI♂, C57♀ and Pygb-KI♀ | mouse | 4 | 8-12 weeks |
| Figure1g | Glycogen measurement | C57♂, Pygb-KI♂, C57♀ and Pygb-KI♀ | mouse | 5 | 8-12 weeks |
| Figura2d~2f | Metabolome profiling analysis | C57 and Pygb-KI | astrocyte | 6 well | / |
| Figure3c~3f | Morphological examination of astrocytes | C57♂, Pygb-KI♂, C57♀ and Pygb-KI♀ | astrocytes | 22  [from 3 mice] | 11 weeks |
| Figure3h~3j | sholl analysis of neuron | C57♂, Pygb-KI♂, C57♀ and Pygb-KI♀ | pyramidal neuron | 19-25  [from 3 mice] | 11 weeks |
| Figure4 | spine analysis of neuron | C57♂, Pygb-KI♂, C57♀ and Pygb-KI♀ | pyramidal neuron | 25-30  [from 3 mice] | 11 weeks |
| Figure5 | Open field test, Elevated plus maze test, Tail suspension test | C57♂, Pygb-KI♂, C57♀ and Pygb-KI♀ | mouse | 12 | 8-12 weeks |
| Figure6a-6i | Open field test, Elevated plus maze test, Tail suspension test | C57 and Pygb-KI | lactating mouse | 7-8 | postpartum days 1-3 |
| Figure6k | Resident-juvenile-intruder test | C57 and Pygb-KI | lactating mouse | 7-8 | postpartum days 1-3 |
| Figure7b | Nest quality assessment | C57 and Pygb-KI | lactating mouse | 20-21 | Day of parturition |
| Figure7d/7f | Nurturing obsevation | C57 and Pygb-KI | lactating mouse | 13-15 | Day of parturition |
| Figure7h/7i | Pup retrieval test | C57 and Pygb-KI | lactating mouse | 10 | Day of parturition |
| Figure7k | IF | C57 and Pygb-KI | lactating mouse | 4 | postpartum day 7 |
| Figure8a/8b | Survival rate of pups | C57 and Pygb-KI | pup | 117 pups (born from C57 dams [n=15]) and 94 pups (born from Pygb-KI dams [n=12]) | PND0 |
| Figure8c | weight of pups | C57 and Pygb-KI | pup | born from 6-8 dams | PND7, PND14, PND21 |
| Figure8e/8f/8g | Separation-induced vocalization | C57 and Pygb-KI | pup | 18 pups (born from C57 dams [n=4]) and 9 pups (born from Pygb-KI dams [n=4]) | PND9 |
| Figure S1 | qPCR | C57 and Pygb-KI | lactating mouse | 4 | postpartum day 7 |
| Figure 9 | qPCR/IF | C57 and Pygb-KI | lactating mouse | 4 | postpartum day 7 |

WB: westing blotting; IF: immunofluorescence; qPCR: Quantitative Real-time PCR
